# CNPY4 is a potential promising prognostic-related biomarker and correlated with immune infiltrates in gliomas

**DOI:** 10.1097/MD.0000000000030044

**Published:** 2022-08-19

**Authors:** Jian-Wen Li, Qian-Rong Huang, Li-Gen Mo

**Affiliations:** a Department of Neurosurgery, Guangxi Medical University Cancer Hospital, Nanning, Guangxi 530021, P.R. China.

**Keywords:** biomarker, CNPY4, glioma, immune infiltrates

## Abstract

Glioblastomas are classified into primary and secondary; primary glioblastomas develop rapidly and aggressively, whereas secondary glioblastomas are more common in grade II and III gliomas. Here, we aimed to demonstrate the role of the CNPY4 gene as a potential biomarker in immune infiltration in gliomas. Based on gene expression profile interaction analysis (GEPIA), we studied the survival model of CNPY4 and evaluated its effect on patients with glioma. The glioma dataset was downloaded from The Cancer Genome Atlas (TCGA) database. Logistic regression was used to analyze the relationship between clinical data and CNPY4 expression. Univariate and multivariate Cox proportional-hazards models were used to compare clinical features and patient survival. The relationship between CNPY4 and immune infiltration in glioma was studied using GEPIA and CIBERSORT online tools. TCGA data were analyzed using gene set enrichment analysis (GSEA). Finally, TIMER was used to analyze the expression and immune infiltration of CNPY4 in glioma to study the cumulative survival rate. Univariate logistic regression analysis showed that increased CNPY4 expression was associated with tumor age, grade, IDH status, and 1p/19q codeletion. Multivariate analysis showed that that downregulation of CNPY4 expression was an independent and satisfactory prognostic factor. CNPY4 expression was correlated with the infiltration level of dendritic cells in glioblastoma. In contrast, in low-grade gliomas, the infiltration level of B cells, dendritic cells, macrophages, neutrophils, and CD4+ T cells was significantly correlated with CNPY4 expression. The GSEA results showed that CNPY4 played an immunoregulatory role in immune-related phenotypic pathways between lymphoid and nonlymphoid cells. The intestinal immune networks for IgA production, rabbit thyroid disease, primary immunodeficiencies, and cancer immunotherapy were enriched by PD-1 blockade. High CNPY4 expression is a biomarker of glioma prognosis and is associated with the immune invasion of glioma.

## 1. Introduction

Gliomas are solid tumors originating from normal glial cells, called astrocytes, oligodendrocytes, and ependymal cells; they have the same histological characteristics as the central nervous system. Gliomas are classified into astrocytomas, oligodendrogliomas, and ependymomas according to different degrees of biological malignancy. The World Health Organization (WHO) classifies gliomas into 4 grades based on their histological characteristics.^[1]^ Grade I is a solid and noninvasive tumor (pilocytic astrocytoma), and Grades II–IV are diffuse invasive gliomas.^[2]^ Grades II and III are low-grade gliomas (LGG), whereas Grade IV is a more invasive high-grade glioma. Grade IV glioma rapidly progresses, is widely invasive, and has high mortality. Grade IV glioma is known as glioblastoma (GBM), which is a widely common and fatal adult brain tumor and can be divided into primary and secondary. Approximately 90% of GBMs are primary GBMs, whereas 10% are secondary. GBM is a highly invasive tumor, common in elderly patients; secondary GBMs are common in young patients with grade II and III gliomas.^[3]^ At present, there are limited methods available for treating glioma, mainly maximum surgical resection within the safe range combined with postoperative radiotherapy and chemotherapy. However, the overall survival time of patients with high-grade gliomas remains short after active treatment.^[4]^ Recently, glioma-related genes have emerged as research hotspots to achieve a breakthrough in glioma treatment.^[5–8]^

In humans, the canopy homolog (CNPY) genes of the canopy fibroblast growth factor (FGF) signaling regulator gene family (CNPY1, CNPY2, CNPY3, and CNPY4) share a conserved cysteine domain of galactose sphingosine-like protein and C-terminal Er recovery sequence.^[9,10]^ CNPY1 is a positive feedback regulator of the fibroblast growth factor (FGF) signaling pathway, which controls progenitor cell aggregation during Kupffer vesicle organogenesis by binding to the extracellular domain of FGFR1 in zebrafish.^[11]^ CNPY2 is regulated by hypoxia-inducible factor-1 alpha.^[12]^ Furthermore, CNPY2 can enhance the proliferation, migration, and tissue angiogenesis of human smooth muscle cells.^[13]^ Upregulated CNPY2 expression can inhibit the activity of p53 in human colorectal cancer, promote tumor growth and angiogenesis, and inhibit apoptosis.^[14]^ CNPY3 and CNPY4 (also known as PRAT4A and PRAT4B, respectively) are combined with Toll-like receptor 4 and Toll-like receptor 1 and regulate their expression on the cell surface and in cellular immune response.^[15,16]^ CNPY4, also known as canopy FGF signaling regulator 4, is a plasma membrane (PM) and endoplasmic reticulum (ER) protein. Only a few studies have demonstrated the relationship between the CNPY4 gene and tumors. Therefore, in this study, we explored the relationship between CNPY4 gene expression and glioma using bioinformatics.

## 2. Materials and Methods

### 2.1. Data collection

We sourced the data for this study from the Cancer Genome Atlas (TCGA). We mainly downloaded 2 types of data for GBM and LGG patients: clinical information and gene expression profiles. Considering that different clinical variables are missing from TCGA samples and the missing situation of different clinical variables is different, there were 634 cases according to the WHO grade and 695 cases as per other clinical variables. To promote research and explore the impact of CNPY4 on the microenvironment of the immune system, we divided tumor tissues into 2 groups according to the expression of CNPY4.

### 2.2. Gene expression profile interaction analysis (GEPIA)

Based on GEPIA (website: http://gepia2.cancer-pku.cn/#index), we analyzed the relationship between CNPY4 expression and the clinicopathological information of GBM and LGG.^[16]^ According to the survival curve of differential expression of the CNPY4 gene produced by GEPIA, in this study, we explored the relationship between gene expression and the prognosis of patients with GBM and LGG. First, CNPY4 expression in different pathological stages was compared using a staging map with pathological stages as variables. Second, a box diagram with tumor status as the variable was constructed to analyze the differentiation of CNPY4.

### 2.3. Tumor immune estimation response (TIMER) analysis

We systematically analyzed the immune infiltration of various cancer types using TIMER.^[17]^ According to gene expression profile, TIMER analysis can determine the number of tumor-infiltrating immune cells (TIICs).^[18]^ Moreover, we analyzed the relationship between CNPY4 expression and immune infiltration density in 32 tumors in the gene module.^[19]^ Typically, such immune infiltration mainly includes B cells, dendritic cells, CD4+ T cells, macrophages, CD8+ T Cells, and neutrophils.

### 2.4. Enrichment analysis

Gene set enrichment analysis (GSEA) is a method to sort genes according to the differences in data between groups of input gene sets to determine their enrichment in different biological functions and signal pathways.^[20]^ Using GSEA, the standardized mRNA datasets of gliomas were separated into 2 categories: a CNPY4 high expression group and a low expression group. In GSEA (4.1.0), we selected “h.all. V7.2. Symbols. GMT” to annotate a gene set, followed by “high expression or low expression” for phenotypic label, and then selected 1000 replacement times. The results showed normalized enrichment score (NES) > 1.5; false discovery rate (FDR) Q < 0.25 and nominal *P*-value (NOM: P) < 0.05 were considered statistically significant.

### 2.5. CIBERSORT immunoinfiltration analysis

CIBERSORT (https://cibersort.stanford.edu/; Alizadeh Lab, Stanford, CA) is used to estimate the gene expression profile. In addition, based on gene expression data, it can evaluate the enrichment of member cell types in mixed cell colonies.^[21]^ First, the standardized mRNA dataset was uploaded to CIBERSORT. The number of permutations was set to 1000 using the web page analysis tool, and the information on the 22 types of immune cells in each sample was obtained. Next, the components of various immune cells between high and low CNPY4 expression groups were compared.

### 2.6. Statistical analysis

We processed the data downloaded from TCGA using R language version 3.5.3. First, we explored the correlation between clinical data and CNPY4 gene expression using a logistic regression algorithm. Next, we analyzed CNPY4 expression and other clinicopathological factors on survival, such as age and sex, using the Cox proportional hazards model with a *P*-value < 0.05. Finally, to reveal the correlation between 22 immune cells, a heat map was constructed.

## 3. Results

### 3.1. Diversification analysis

**Figure 1.**
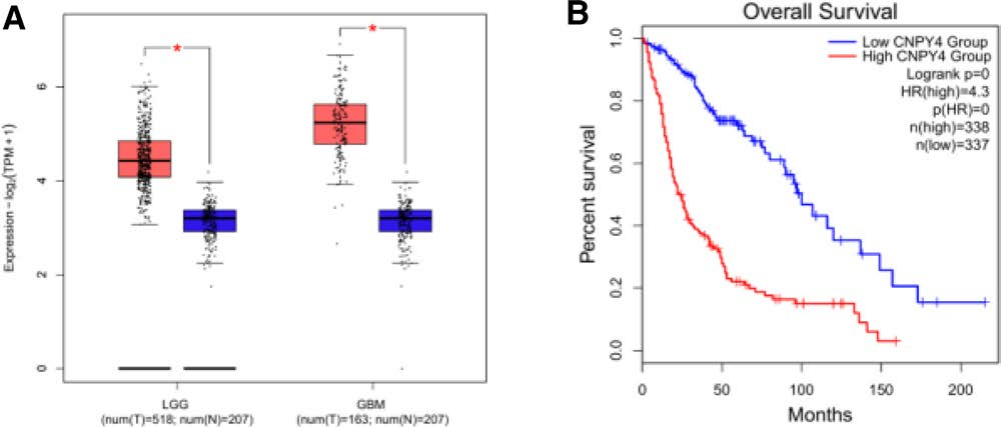
(A). Differential expression of CNPY4 in different disease state (Tumor or Normal). (B). Survival curve of differential CNPY4 expression were analyzed by GEPIA.

**Figure 2.**
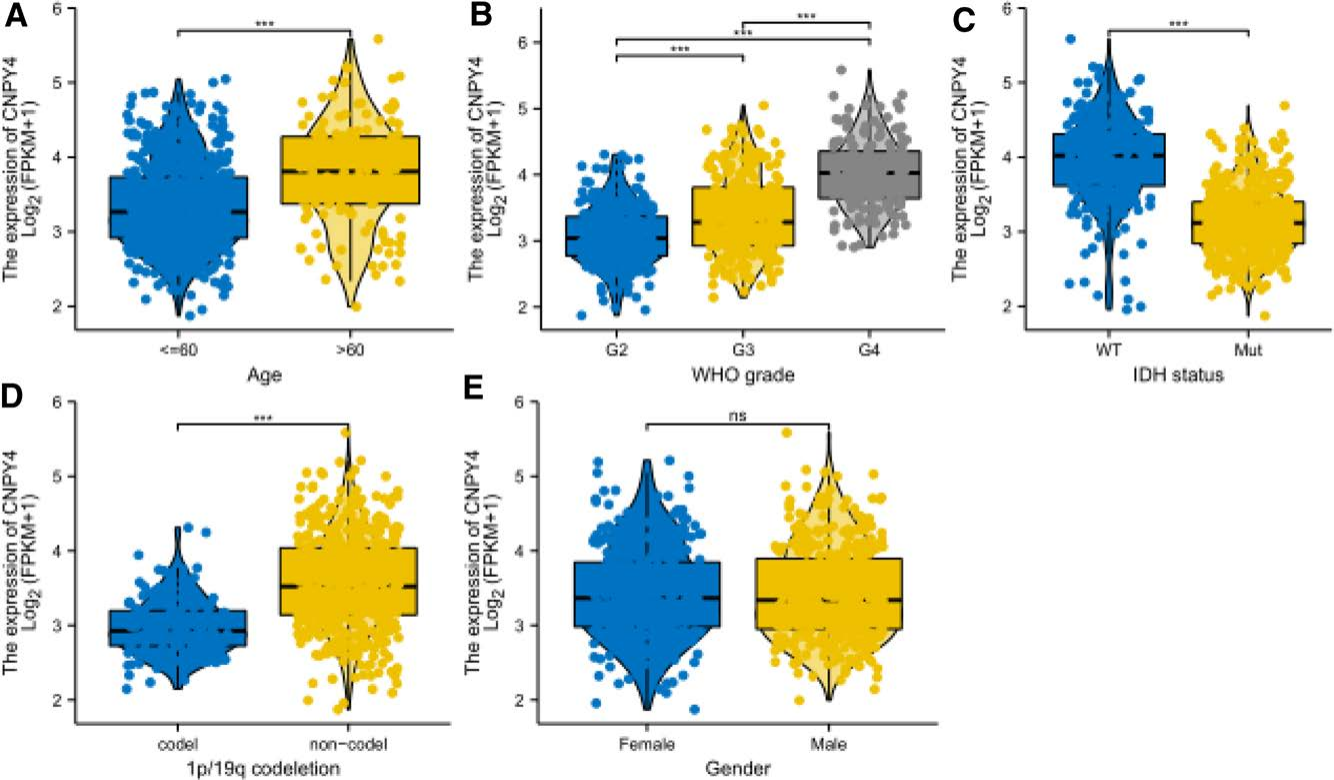
Correlation analysis between CNPY4 expression and clinical characteristics using the TCGA database. Differential expression of CNPY4 was significantly related to (A) age (≤60, n *=* 553; >41, n = 143); (B) grade (WHO II, n = 224; WHO III, n = 243;WHO IV, n = 168); (C) IDH status (Mutant, n *=* 440; Wildtype, n = 246); (D) 1p19q codeletion (Codel, n *=* 171; noncodel, n *=* 518), and (E) gender (female, n *=* 298; male, n *=* 398).

**Figure 3.**
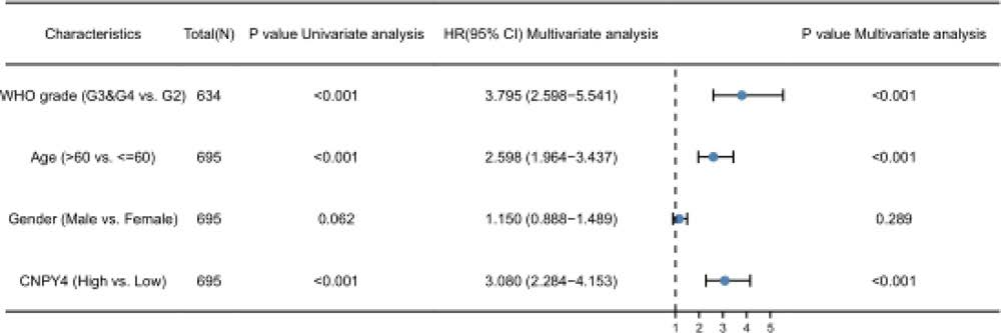
Multivariate Cox analysis of CNPY4 expression and other clinical pathological factors (as age, tumor grade, and CNPY4 expression are independent prognostic factors).

The overall survival, expression on the box plot, and pathological stage plot were obtained based on GEPIA (Figs. 1 and 2). The expression of CNPY4 in GBM and LGG was significantly higher than that in other undiagnosed brain tissues (Fig. 1A). Prognostic analysis revealed that in the CNPY4 low expression group, the survival time was significantly higher than that in the high expression group (*P* < .05, Fig. 1B). Univariate Cox proportional-hazards regression model showed that the grades significantly correlated with the overall survival rate (Table 1). Using multivariate analysis, age, neoplasm grade, and CNPY4 expression were confirmed to be independent prognostic factors (Fig. 3). In summary, CNPY4 expression was considered related to the neoplasm grade with the increase in tumor size.

**Table 1:** Univariate and multivariate Cox analysis of CNPY4 and clinicopathological parameters.

Characteristics	Total (N)	HR (95% CI) Univariate analysis	*P* value univariate analysis	HR (95% CI) multivariate analysis	*P* value multivariate analysis
WHO grade (G3&G4 vs G2)	634	5.642 (3.926–8.109)	<0.001	3.795 (2.598–5.541)	<0.001
Age (>60 vs ≤60)	695	4.668 (3.598–6.056)	<0.001	2.598 (1.964–3.437)	<0.001
Gender (Male vs female)	695	1.262 (0.988–1.610)	0.062	1.150 (0.888–1.489)	0.289
CNPY4 (High vs Low)	695	4.296 (3.271–5.642)	<0.001	3.080 (2.284–4.153)	<0.001

### 3.2. Relationship between CNPY4 gene expression and immunity

**Figure 4.**
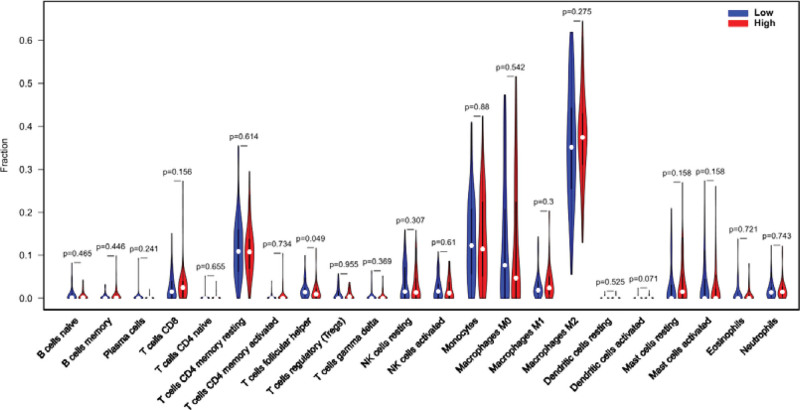
The proportion of 22 subpopulations of immune cells T cell follicular helper (*P* = .049), which was higher in the high expression group than in the low expression group.

Tumor-infiltrating lymphocytes are independent predictors of sentinel lymph node status and survival in cancer patients.^[21]^ Therefore, we used CIBERSORT on the downloaded data to reveal the gene expression profile in GBM and LGG, revealing the relationship between CNPY4 expression and immune infiltration. We compared the abundance of 22 immune cells in 2 groups (high and low expression group) (Fig. 4) and found that based on the proportion of 22 immune cell subsets, the major immunocytes affected by CNPY4 expression were resting natural killer (NK) cells, activated NK cells, and M2 macrophages. The proportion of resting NK cells in the high expression group (*P* = .018) was significantly higher than that in the low expression group. Moreover, the proportion of activated NK cells (*P* = .008) and M2 macrophages was reduced in the high expression group (*P* = .034) compared with the low expression group.

### 3.3. Cumulative survival study

**Figure 5.**
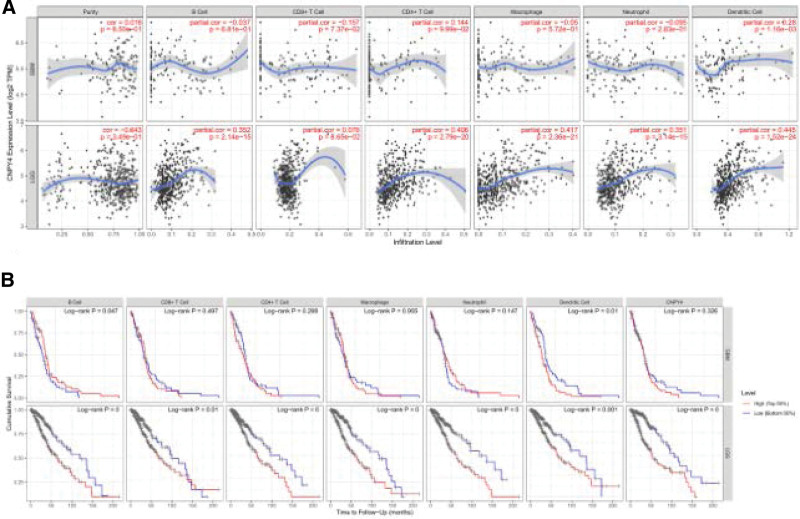
(A). CNPY4 expression level has significant correlations with infiltrating levels of dendric cell infiltration in GBM was positive correlation (Partial Cor = 0.28). In LGG, the logarithm of CNPY4 expression level was positively bound up with the infiltration levels of B cells (Partial Cor = 0.3520, CD4+ T cells Partial Cor = 0.406), macrophages (Partial Cor = 0.417), neutrophils (Partial Cor = 0.351) and dendritic cells (Partial Cor = 0.445) in tumor tissues (*P* < .05). (B). Cumulative survival of CNPY4 in GBM and LGG. The results showed that 6 immune cells are B cells, T cells, Macrophages, Neutrophils and Dendritic cells) and CNPY4 were the survival indexes of LGG patients. In GBM patients, only B cell and dendritic cell were related to survival.

Tumor immune infiltration is a key factor in tumor progression and significantly affects the survival rate of tumor patients.^[22]^ Therefore, in various cancer classifications, we used TIMER to study the relationship between CNPY4 expression and immune infiltration levels. The CNPY4 expression degree was associated with poor prognosis and high immune infiltration of GBM and LGG. TIMER analysis showed that CNPY4 was positively correlated (Partial Cor = 0.28) with the infiltration levels of dendric cells in GBM. In LGG, the logarithm of CNPY4 expression was positively correlated with the infiltration levels of B cells (Partial Cor = 0.3520) CD4+ T cells (Partial Cor = 0.406), macrophages (Partial Cor = 0.417), neutrophils (Partial Cor = 0.351), and dendritic cells (Partial Cor = 0.445) in tumor tissues (*P* < .05) (Fig. 5A).

Simultaneously, we explored the cumulative survival of CNPY4 in GBM and LGG tissues and found that B cells, T cells, macrophages, neutrophils, dendritic cells and CNPY4 were the survival indices for LGG patients, whereas, in GBM patients, only B cells and dendritic cells were related to survival (Fig. 5B).

### 3.4. Enrichment analysis

**Figure 6.**
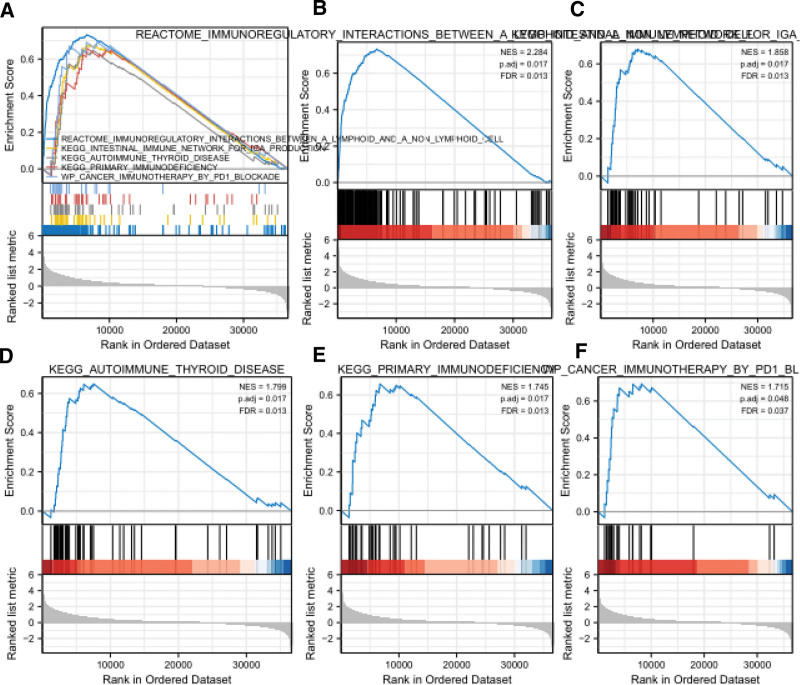
Enrichment plots from gene set enrichment analysis (GSEA). Covers autoimmune thyroid disease, immune regulatory interaction between lymphocytes and nonlymphocytes, and intestinal immune network producing IgA, Primary immunodeficiencies, Cancer immunotherapy by PD-1 blockade pathway.

Using GSEA, we identified immune-related signaling pathways involved in glioma with high and low expression of CNPY4. Cumulative survival of CNPY4 in GBM and LGG. The results showed that 6 immune cells are B cells, CD4+ and CD8+T cells, Macrophages, Neutrophils and Dendritic cells) and CNPY4 were the survival indexes of LGG patients. In GBM patients, only B cell and dendritic cell were related to survival. In LGG, 6 immune cells are all related to survival (Fig. 6).

## 4. Discussion

CNPY4, a member of the CNPY gene family, is generally expressed at high levels in the testis, thymus, gastrointestinal tract, and spleen.^[23]^ However, only limited studies on other aspects have been conducted. Therefore, in the present study, we investigated the potential role of CNPY4 in glioma and analyzed its expression in human glioma patients using TCGA and RNA-seq databases. CNPY4 mRNA expression was correlated with the tumor in glioma patients. In addition, we revealed that CNPY4 expression was associated with the prognosis of glioma, and the downregulation of CNPY4 expression was an independent and satisfactory prognostic factor. Moreover, we found that different degrees of immune infiltration and immune marker sets were related to CNPY4 expression in gliomas.

First, we used the GEPIA online database to reveal that CNPY4 expression was associated with the survival of glioma patients. In addition, the downregulation of CNPY4 expression facilitated the prognosis of patients. We downloaded the dataset from TCGA to reveal the expression mechanism and relationship of CNPY4 in tumors. Next, we analyzed TCGA data using R software (v3.5.3), revealing that CNPY4 expression was associated with tumor grade, age, IDH status, and 1p/19q codeletion. More importantly, multivariate regression analysis suggested that CNPY4 expression was an independent prognostic factor in glioma patients. CIBERSORT revealed that CNPY4 expression was positively correlated with the proportion of dendritic cells in gliomas. Based on these findings and results from previous studies, the relationship between CNPY4 expression and glioma immune infiltration can be studied. Analyzing the genes in different immune cells and CNPY4 expression, we clarified that CNPY4 markedly influences the regulation of the tumor immune microenvironment. We believe that CNPY4 can eventually be used as an effective tumor biomarker.

The immune checkpoint inhibitors have achieved lasting effects in various tumors.^[24]^ The treatment of tumor patients may be related to the quantity and distribution of TIICs, and consequently, TIICs have become a potential drug target to improve the survival of such patients.^[25]^ The immune score proposed considering tumor immune characteristics has emerged as a new method to determine tumor prognosis, mainly based on evaluating T cell subsets, especially CD3+ T cells and CD8+ T cells.^[26]^ The occurrence and development of cancer are closely related to the abnormal activation of proto-oncogenes and tumor suppressor genes. The human B-cell lymphoma factor 3 (BCL-3) gene, a proto-oncogene, is located on chromosome 19q13, which is mainly involved in regulating cell proliferation and apoptosis. Abnormal expression of BCL-3 was first observed in chronic B-cell lymphocytic leukemia.^[27]^ BCL-3 expression has been found to be upregulated in various solid tumor tissues, promoting tumor cell proliferation.^[28–30]^ High BCL-3 expression can inhibit breast cancer cell apoptosis and participate in the occurrence and development of breast cancer.^[31]^ Among all evaluated tumor types, the expression of genes associated with T cells and B cells (including BCR fragments) was highly correlated.^[32,33]^ In different immune cell types, the expression of immune markers is strongly correlated, showing diversity; however, in the most predictable tumor immune infiltration, B cells support antitumor immune response.^[34,35]^ Therefore, T cells and B cells have high density, helping CNPY4 have a favorable effect on glioma treatment.

To reveal the mechanism underlying the role of CNPY4 in glioma, GSEA was used to explore immune-related pathways. The results showed that immunoregulatory interactions between lymphoid and nonlymphoid cells, the internal immune network for IgA production, autoimmune thyroid disease, primary immunodeficiencies, and cancer immunotherapy by PD-1 blockade were differentially enriched due to CNPY4 overexpression. CNPY4 may be an immune prognostic index and therapeutic target of glioma. Moreover, we revealed a favorable relationship between CNPY4 expression and dendric cell infiltration in GBM (Partial Cor = 0.28). In LGG, the logarithm of CNPY4 expression was favorably correlated with the infiltration degrees of B cells (Partial Cor = 0.352), CD4+ T cells (Partial Cor = 0.406), macrophages (Partial Cor = 0.417), neutrophils (Partial Cor = 0.351), and dendritic cells (*P* < .05, Partial Cor = 0.445). Furthermore, we showed the important effect of CNPY4 on dendritic cell immune infiltration in glioma. At any rate, this barrier to thinking should be considered. First, due to the limitations of these open databases, some important clinical parameters, such as tumor resection degree, tumor measurement and area, and preoperative status, may affect the high expression of CNPY4. In addition, in this study, the in silico expression profile findings of CNPY4 have not been validated in vitro or in vivo, transcript levels are only a representation and not a confirmation of protein levels. Reexamination is usually required, and some of the characteristics and expression of CNPY4 need to be confirmed by necessary tests and an extensive clinical cohort.

## 5. Conclusion

In the present study, we demonstrated the relationship between CNPY4 and gliomas. The findings of this study revealed that CNPY4 is a key gene in gliomas that might serve as a prognostic biomarker, and CNPY4 expression may be used to investigate immune infiltration in glioma patients. However, due to sample limitations and lack of internal and external data validation, more studies and experiments are necessary to verify the effectiveness of these predictors. Furthermore, additional studies are needed to analyze the mechanisms underlying the pathophysiological role of CNPY4 in gliomas.

MD-D-21-06041

## Author contributions

Conceptualization: Jian-Wen Li, Li-Gen Mo.

Data curation: Qian-Rong Huang, Jian-Wen Li.

Formal analysis: Jian-Wen Li, Qian-Rong Huang.

Methodology: Qian-Rong Huang, Jian-Wen Li.

Resources: Jian-Wen Li, Qian-Rong Huang.

Software: Qian-Rong Huang, Jian-Wen Li.

Visualization: Qian-Rong Huang, Li-Gen Mo.

Writing—original draft: Jian-Wen Li, Qian-Rong Huang.

Writing—review & editing: Li-Gen Mo.
